# Oral Care Associated With Less Microaspiration in Ventilated Cardiac Patients

**DOI:** 10.1097/PG9.0000000000000290

**Published:** 2023-02-10

**Authors:** Nishant Patel, Philip Lin, Michael Stack, Janet M. Conrad, Harun Fakioglu, Bassam Abomoelak, Karoly Horvath, Devendra I. Mehta

**Affiliations:** From the * Center for Digestive Health and Nutrition, Arnold Palmer Hospital for Children, Orlando, FL; † Department of Surgery, The Pennsylvania State University, College of Medicine, Hershey, PA; ‡ Department of Pediatric Cardiology Arnold Palmer Hospital for Children, Orlando, FL; § Pediatric Gastroenterology and Translational Research Laboratory, Cardiology Arnold Palmer Hospital for Children, Orlando, FL.

**Keywords:** microaspiration, aspiration, oral care, pepsin A, pepsin C, ventilated, intubated

## Abstract

Aspiration is common in mechanically ventilated patients and may predispose patients to aspiration pneumonia, chemical pneumonitis, and chronic lung damage. Pepsin A is a specific marker of gastric fluid aspiration and is often detected in ventilated pediatric patients. We investigated the effect of oral care and throat suctioning in the detection of pepsin A in tracheal aspirates (TAs) up to 4 hours after these procedures.

**Methods:**

Twelve pediatric patients between age 2 weeks to 14 years who underwent intubation for cardiac surgery were enrolled in this study. Six of the 12 patients were consented before their surgery with initial specimen collected at the time of intubation and last one shortly before extubation (intubation duration < 24 hours). The remaining 6 patients were consented after cardiac surgery. All specimens were collected per routine care per respiratory therapy protocol and shortly before extubation (intubation duration > 24 hours). Tracheal fluid aspirates were collected every 4 to 12 hours in the ventilated patients. Enzymatic assay for gastric pepsin A and protein determination were performed. The time of oral care and throat suctioning within 4 hours prior was recorded prospectively.

**Results:**

A total of 342 TA specimens were obtained from the 12 intubated pediatric patients during their course of hospitalization; 287 (83.9%) showed detectable total pepsin (pepsin A and C) enzyme activity (> 6 ng/mL) and 176 (51.5%) samples had detectable pepsin A enzyme levels (>6 ng/mL of pepsin A). Only 29 samples of 76 samples (38.2%) had evidence of microaspiration after receiving oral care, while 147 of 266 (55.3%) samples were pepsin A positive when no oral care was provided. Odds ratio is 0.50 (Cl 0.30–0.84), and the number needed to treat is 5.8 (Confidence interval 3.4–22.3). Testing air filters for pepsin was not beneficial.

**Conclusion:**

Oral care is a highly effective measure to prevent microaspiration of gastric fluid in ventilated pediatric patients. The number needed to treat (5.8) suggests this is a very effective prevention strategy. Our study suggests that pepsin A is a useful and sensitive biomarker that allows identification of gastric aspiration.

**What Is Known**

- Prolonged ventilation increases the risk of aspiration.
- Pepsin assays have emerged as promising markers for aspiration.
- The benefits of oral care in ventilated adult patients are well-studied, but its utility in ventilated pediatric populations is less clear.

**What Is New**

- Pepsin A assays are gastric-specific and more useful than total pepsin assays in identifying aspiration of gastric contents.
- Routine oral care is associated with decreased aspiration in this ventilated pediatric population.
- Standardization of oral care in ventilated pediatric patients may decrease rates of aspiration and associated complications.

## INTRODUCTION

Mechanically ventilated patients are at risk for aspiration and prolonged ventilation may predispose patients to aspiration pneumonia, chemical pneumonitis, and chronic lung damage ^(1–3).^ The pathomechanism of aspiration associated with mechanical ventilation has been well- documented, as the endotracheal tube (ETT) suppresses the cough and mucociliary response while creating accessory paths for gastric contents and bacteria from the upper respiratory tract to the lower respiratory tract ^(4,5).^ Many associated risk factors influence the prevalence and outcomes of aspiration in ventilated patients, such as age of the patient, weight, type and location of the surgery, surgical complications including but not limited to recurrent laryngeal nerve injury, congenital anomalies, gastroesophageal reflux (GER), supine positioning, onset and method of feeding, ventilator settings, and duration of intubation ^(6–8)^. While cuffed ETTs in mechanically ventilated children were implemented to minimize some of the risks of aspiration, microaspiration may still occur ^(9,10).^ Of note, patients undergoing surgery for congenital heart disease are at increased risk for aspiration as vocal cord dysfunction and dysphagia are common postoperative complications ^(11,12).^

To reduce the occurrence of ventilator-associated pneumonia (VAP) and other pulmonary manifestations of gastric aspiration, a noninvasive clinical assay that documents active microaspiration is required. The 24-hour esophageal pH probe and multiple intraluminal impedance (MII) study are commonly used to aid in diagnosis of gastroesophageal reflux (GER). Esophageal MII measures the impedance of luminal contents between several electrodes contained in the catheter allowing detection of all reflux episodes and their anatomic characterization. However, these modalities can be invasive, expensive, and unreliable in neonate populations ^(13–15).^ The use of methylene blue, glucose, and lactose assays to detect aspiration are unreliable ^(16–19).^ The traditional method to detect microaspiration is the lipid-laden macrophage test, which stains bronchoalveolar fluid obtained by bronchoscopy and demonstrates the presence of lipid-laden macrophages ^(20,21)^. A study by Krishnan et al ^(20)^ concluded that the lipid-laden macrophage test performed on tracheal aspirates (TAs) from mechanically ventilated patients is not a sensitive or specific marker for microaspiration and promoted the use of the pepsin assay. Pepsin has emerged as a promising biomarker for aspiration, and assays have been developed to test airway samples including pharyngeal swabs, middle ear fluid, tracheal and bronchoalveolar washing fluids for gastric pepsin ^(22–25).^

Oral care is a widely accepted practice utilized in intensive care units, although there is institutional variation in its definition and application. Care of ventilated patients can also include ETT and oral suctioning. Deep oral or throat suctioning can remove secretions and fluid accumulation in the pharynx. Oral care usually consists of an oral rinse with antiseptic solution or saline and use of a suction system ^(26).^ In adult populations, oral suctioning prior to position change and continuous oral suctioning have been associated with reduced incidence of VAP ^(27,28).^ Fewer studies investigate the utility of these interventions in ventilated pediatric patients than adult populations. A recent Cochrane analysis found inadequate evidence to determine the optimal frequency of ETT suctioning in ventilated neonates to prevent respiratory morbidity ^(29).^ The presence of salivary pepsin A has been correlated with clinical GER in preterm neonates ^(30).^ Studies examining the effect of oral care interventions on VAP in pediatric populations report mixed results ^(31,32)^.

The aim of this study was to assess the effect of oral care on microaspiration in ventilated pediatric patients following cardiac surgery. Measurement of TA total pepsin and pepsin A enzyme activity were utilized to explore correlation with age, acid suppressive medication, ETT suctioning, duration of intubation, and patient positioning. We hypothesized that oral care is associated with less microaspiration.

## PATIENTS AND METHODS

### Study Subjects

Infants and children who were intubated for cardiac surgery and were admitted to the cardiac intensive care unit at Arnold Palmer Hospital in Orlando, Florida, were enrolled in this study over a 4-year period. Caregivers signed an Institutional Review Board approved informed consent form prior to enrollment into the study. Patients enrolled did not have pathological reflux activity.

### Patient Clinical Data Collection

Nursing and respiratory therapy staff collected data pertinent to the study. These included collection date and time, ETT cuff status, onset and type of feeds, position of the patient (prone, lateral decubitus, supine) and medications (proton pump inhibitor or H2 receptor blocking agent).

### Oral Care and Suction Procedures

Oral care consisted of Yankauer suction upon visual pooling of oral secretions and oral saline rinse. No oral antiseptic rinse was used. ETT suction was performed by respiratory therapists. A suction catheter down to the end of the ETT, and approximately 2 mL of physiologic saline was administered, and gentle suction was applied. Throat suctioning included insertion of Yankauer and/or red rubber catheter and suction of oropharynx and hypopharynx.

### Sample Collection Procedures

Collections of the tracheal washing fluid were obtained by the standard protocol and technique used by the respiratory therapists. A suction catheter was passed down to the end of the ETT, and approximately 2 mL of physiologic saline was administered, and gentle suction was applied. The respiratory secretions were collected into sterile Lukens tube and attached to a sterile suction catheter. The TA samples were obtained before, during, and up to twice daily after surgery until the patients were extubated. The TA samples were collected and stored at −20 °C until the pepsin assay.

### Pepsin Assay

The enzymatic assay initially described by Krishnan et al (18) was modified to increase the lower limit of sensitivity from 250 to 6.0 ng/mL. TAs were thawed on ice and centrifuged to remove debris before assay. Pepsin (porcine) standards (6.0–400 ng/mL) were prepared in saline containing 0.1 mg/mL bovine serum albumin (BSA). Gastric fluid obtained during routine endoscopy from unidentified individuals was used as positive control for the assay. Gastric fluid was diluted 500× using the saline/BSA buffer. TA samples, standards, and controls (50 µL) were incubated with 23 µL of 129 mM hydrochloric acid on ice for 15 minutes to inactivate lysosomal acid hydrolase (cathepsin D) and to convert pepsinogen to active pepsin.

Next, samples were incubated with 20 µL of 0.5% fluorescein isothiocyanate (FITC) casein type III, (Sigma-Aldrich, St. Louis, MO) for 3 hours at 37°C. The enzymatic reaction was stopped by precipitation with 20% ice-cold tri-chloroacetic acid and 1.2 mg/mL BSA for 20 minutes, which was followed by centrifugation. The supernatants (38 µL) were transferred to a microplate and mixed with 212 µL of 500 mM Tris buffer at pH 8.5. The micro-plates were read in a spectrofluorometer (Cytofluor Plate Reader 4000, Perseptive Biosystem, Stafford, TX) with excitation at 485 nm and emission at 530 nm. The net fluorescent intensity was calculated by subtracting values from blanks in which pepsin was inactivated by heat prior to the addition of the substrate. The final pepsin concentration of a sample was determined based on the net fluorescent intensity of the known concentrations of the standards. Due to variable dilution of the specimen during collection, qualitative measurement of pepsin A was utilized. The empirical pepsin level differentiating aspirates positive or negative for pepsin was set at the lower limit of the sensitivity of the assay, 6.0 ng/mL. The presence of pepsin A above 6 ng/mL is abnormal and indicative of aspiration. Gastric pepsin levels in gastric fluid from two unidentified children were 170 and 220 ng/mL, respectively. In addition, we also measured pepsin in sera; pepsin was not detected in control sera.

## DATA COLLECTION AND ANALYSIS

Clinical data were collected prospectively and recorded in Excel sheets. Data analysis was performed by the chi-square test for binary variables. Statistical analysis was conducted using the Statistical Package for the Social Sciences (SPSS v.24.0: IBM; Chicago, IL).

## RESULTS

We enrolled 12 pediatric patients who underwent a cardiac surgery for underlying cardiac anomaly. Six (patients 7–12) were consented before their cardiac procedure, and initial samples were collected at the time of intubation. All 6 patients were extubated within 48 hours before initiating feeds. Patients 16 were consented after their cardiac procedure. The female:male ratio of our sample was 2:1. The majority of the patients were <6 months of age (n = 8), and the oldest child was 14 years of age at the time of the surgery. Patients’ demographic characteristics, congenital cardiac anomalies, use of acid suppressive medication, and duration of intubation are summarized in Table 1.

**TABLE 1.** Patient demographics and characteristics of the congenital heart disease, duration of intubation postoperative, history of acid suppressive medication

Patient	Age (mo)	Sex	Congenital cardiac anomaly	Duration of intubation (d)	Acid suppressive medication
1	0.5	F	Shone Complex	26	Ranitidine
2	168	F	Atrioventricular canal defect	8	Omeprazole
3	0.75	F	CoA	6	Ranitidine
4	4	M	ASD	1	Ranitidine
5	4	M	VSD	41	Ranitidine
6	3	F	DOVR	40	Ranitidine
7	0.5	F	ASD and VSD	<1	Ranitidine
8	1	F	Tetralogy of Fallot	2	Ranitidine
9	34	M	TGA, Single Ventricle and CoA	<1	Ranitidine
10	4	M	DOVR and VSD	2	Ranitidine
11	6	F	TGA and Pulmonic Valve stenosis	<1	Ranitidine
12	8	F	ASD, VSD, and PDA	2	Ranitidine

Congenital cardiac anomalies. ASD = atrial septal defect; CoA = coarctation of the aorta; DORV = double outlet right ventricle; PDA = patent ductus arteriosus; TGA = transposition of the great arteries; VSD = ventricular septal defect.

A total of 342 TAs were obtained from the 12 patients during their course of hospitalization. Of the 342 aspirates, 287 (83.9%) showed detectable total pepsin (pepsin A and C) enzyme activity (>6 ng/mL), and 176 (51.5%) samples had detectable pepsin A enzyme levels (>6 ng/mL). Eleven patients were on acid suppressive medication (Table 2).

**TABLE 2.** Results: Tracheal aspirate samples, total pepsin and pepsin A, pepsin A positive samples with respect to position collected, and endotracheal tube type

Patient	Number of TA Samples	Number of Pepsin A+ samples	Number of total pepsin+ samples	Pepsin A+/total pepsin+ (%)	Cuffed ET tube (yes/no)
1	43	23	39	56.4	Yes
2	21	6	14	42.8	Yes
3	13	11	13	84.6	Yes
4	3	2	3	66.7	No
5	88	27	72	36.1	Yes
6	151	89	125	71.2	Yes
7	3	3	3	100.0	Yes
8	5	4	5	80.0	Yes
9	2	2	2	100.0	yes
10	6	4	4	100.0	yes
11	3	3	3	100.0	yes
12	4	4	4	100.0	yes
Study totals (%)	342	176 (51.5)	287 (83.9)	78.2	11/12

Pepsin A + = Pepsin A enzyme level > 6 ng/mL; suggestive of microaspiration. ET = endotracheal; Lateral d = lateral decubitus; TA = tracheal aspirate.

The role of acid suppressive medications and cuffed ETT were not able to be evaluated, as these variables were utilized in nearly all patients. Pepsin was not detected in any of the ventilator exhaust filter derived samples, even after concentrating with dialysis. Aspiration was noted in sample cases where nutrition support (total parenteral nutrition, nasojejunal, or continuous gastric feeds) was initiated.

Oral care was performed as part of the routine care by the bedside nurse at least once a shift (8 hours) and then as needed. Tracheal suctioning was performed by the respiratory therapist at least once a shift and more frequently if needed. TA pepsin A enzyme activity of samples collected within 4 hours of oral care were compared to samples that were not collected after oral care (>4 hours after oral care). A number of ETT and throat suctioning were recorded. Qualitative measurements of pepsin A positive and negative samples were performed as described previously. Only 29 of 76 samples (38.2%) had evidence of microaspiration after receiving oral care, while 147 of 266 (55.3%) samples were pepsin A positive when no oral care was provided. Odds ratio is 0.50 (Cl 0.30–0.84) and the number needed to treat is 5.8 (Confidence interval 3.4–22.3) (Tables 3 and 4).

**TABLE 3.** Oral care, endotracheal tube, and throat suctioning

Patient	Oral care	Endotracheal tube suctioning	Throat suctioning	Pepsin A+ samples after oral care*	Pepsin A− samples after oral care*	Duration of intubation (d)
1	21	22	6	9	11	26
2	1	6	0	1	8	8
3	6	15	6	4	3	6
4	3	3	0	1	2	1
5	5	20	9	7	9	4
6	12	68	7	7	14	40
7	0	3	0	0	0	<1
8	0	5	0	0	0	2
9	0	2	0	0	0	<1
10	2	6	2	0	2	2
11	1	3	1	0	1	<1
12	0	4	1	0	0	2

*Samples collected within 4 hours of receiving oral care.

**TABLE 4.** Oral care association with microaspiration

	Microaspiration				
		(+)	(−)	Totals	
	(+)	29	47	76	
Oral care	(−)	147	119	266	*P* value < 0.0092
	Totals	176	166	342	Risk ratio = 0.6905
					Odds ratio = 0.4995

Pepsin A+ = Pepsin A enzyme level > 6 ng/mL; suggestive of microaspiration. Pepsin A− = Pepsin A enzyme level < 6 ng/mL; not suggestive of microaspiration.

## DISCUSSION

Microaspiration frequently occurred in our patients following surgery for congenital heart disease. This study found that oral care had a significant protective effect from microaspiration in mechanically ventilated pediatric patient. This is one of the first studies to utilize TA pepsin A enzyme activity to evaluate the effects of oral care on mechanically ventilated children.

Total pepsin and gastric-specific pepsin A activity were assessed in 342 TA samples to detect aspiration of gastric contents. Pepsin is present in different isoforms including pepsin A and pepsin C. Pepsin A is uniquely present in the stomach, while pepsin C has been found in lung tissue and expressed by type II pneumocytes ^(33,34).^ The detection methods currently used such as dye studies, glucose and lactose assays and lipid-laden macrophages for detection of gastric aspiration have poor sensitivity and specificity ^(16,17,35–37).^ Most prior studies involving mechanically ventilated pediatric patients utilized total pepsin to detect aspiration of gastric contents ^(22,24,38).^ However, more recent studies differentiated pepsin isoforms A and C in tracheal secretions and questioned the validity of nonspecific pepsin assays ^(39,40).^ In our study, pepsin A was measured qualitatively to differentiate samples demonstrating microaspiration activity. We believe that variability in specimen dilution during collection at the bedside makes quantitative measurement of pepsin A activity less reliable for interpretation. Our study found that 83.9% of total samples were positive for total pepsin, whereas 51.5% of samples were positive for pepsin A.

These findings support the need for a gastric-specific assay to confirm presence of pulmonary aspiration of gastric contents. Future study of TA pepsin A activity alongside multichannel intraluminal impedance (MII) evaluation may be valuable to confirm our findings.

Oral care typically consists of an oral rinse with antiseptic solution or saline and use of a suction system. However, no standard of care is generally accepted for oral hygiene in ventilated pediatric patients, and practices vary widely by institution. Oral care at the bedside also varies widely, and can be affected by individual performance, staffing, and quality or lack of training ^(26).^ Oral application of chlorhexidine decreases nosocomial respiratory infections in adult populations prior to heart surgery ^(41).^ Studies examining the effect of oral care interventions on VAP in pediatric populations show varying results ^(31,32).^ No prior studies examine the effect of oral care on aspiration of gastric contents in mechanically ventilated pediatric patients.

Our study found decreased microaspiration in samples after oral care intervention (19.0%) compared with samples from patients with no oral care (56.0%). We believe that routine oral care and suctioning of oropharynx results in decreased volume of potential pulmonary aspirate accumulated in the oral cavity and pharynx. Aspirate content of premature neonates was found to travel to the proximal esophagus or pharynx during the majority of reflux episodes ^(42).^ This supports our claim that management of oropharyngeal fluid collections has important clinical utility in the prevention of aspiration in mechanically ventilated pediatric patients.

Esophageal MII can detect all reflux episodes and their proximal extent. Evaluation of MII in conjunction with TA pepsin A activity would facilitate the accurate quantification of each reflux episode and provide anatomic characterization of each episode with respect to proximal extent of gastric contents. Recently, salivary pepsin A was associated with GER episode in pediatric patients undergoing MII probe monitoring ^(43).^ Further studies evaluating salivary pepsin A in relation to TA pepsin A and MII/pH studies are warranted to explore the utility of biomarker pepsin A to detect microaspiration. The effect of oral care on clinical outcomes in this patient population is not well understood, and further study of this nonpharmacologic and simple intervention is warranted.

Gastric and postpyloric feeding in infants can affect the incidence of aspiration and associated pneumonias. Some studies have found that postpyloric feeds in preterm neonates can reduce the incidence of aspiration pneumonia while others have shown no clear benefit ^(44,45).^ However, benefits of postpyloric feedings can vary between individual patients ^(46,47).^ Similarly, our study showed varied pepsin activities in relation to feeds.

A limitation of this study is the inherent variability in specimen dilution during collection at the bedside leading to the qualitative measurement of pepsin A as opposed to a quantitative measurement. Another limitation includes the small sample size and absence of gold standard diagnostic modality such as a pH or MII study.

Oral care decreases microaspiration of gastric fluid in ventilated pediatric patients. The number needed to treat (5.8) suggests this is a very effective strategy. Our study suggests that pepsin A is a useful and sensitive biomarker that allows identification of gastric aspiration.
